# The Influence of Entrepreneurial Psychological Leadership Style on Organizational Learning Ability and Organizational Performance

**DOI:** 10.3389/fpsyg.2020.01679

**Published:** 2020-07-23

**Authors:** Yixu Tong

**Affiliations:** School of Economics and Management, Tsinghua University, Beijing, China

**Keywords:** organizational learning ability, transactional psychology, transformational psychology, multiple regression model, entrepreneurial psychology leadership style

## Abstract

In order to study the influence of different entrepreneurial psychological leadership styles on organizational learning ability and organizational performance and to provide theoretical basis for the improvement of organizational benefits of entrepreneurial enterprises in the future, 421 general managers, middle managers, and grassroots managers of 350 small- and medium-sized private enterprises in Beijing were surveyed by questionnaire in two forms: online and on-site. Then, a hypothesis model of the relationship between different entrepreneurial psychological leadership styles and organizational learning ability and organizational performance was constructed. The correlation between transformational, transactional, and laissez-faire psychological leadership styles and organizational performance and organizational learning ability was analyzed using multiple regression models. AMOS 7.0 software was used to simulate and verify the model. The results show that transformational psychological leadership style is positively correlated with organizational learning ability, financial performance, and growth performance; transactional psychological leadership style is positively correlated with organizational financial performance, growth performance, and organizational learning ability; there is no correlation between laissez-faire psychological leadership style and organizational financial performance and growth performance, but there is a significant positive correlation between laissez-faire psychological leadership style and organizational learning ability. The covariance between the error terms of the internal and external potential variables of the model is positive, and the factor load value of each potential variable and the observed variable is between 0.5 and 1, which shows that transformational and transactional psychological leadership styles have a more positive influence on organizational financial performance and learning ability than a laissez-faire psychological leadership style. The hypothesis model of the relationship between entrepreneurial psychological leadership styles and organizational learning ability and organizational performance conforms to the adaptation criteria and is feasible.

## Introduction

Entrepreneurship is a process in which entrepreneurs optimize and integrate their own resources or make efforts to create greater economic or social value. It can not only realize self-employment and complete individual ideas but also play an important role in economic development and the expansion of social work positions (Laguía et al., 2019; Saebi et al., 2019; Darwish et al., 2020). Organizational performance refers to the quantity, quality, efficiency, and profitability of organizational tasks completed in a certain period of time, which is the core issue of strategic management research, and how to effectively improve organizational performance is naturally the most concerned issue for entrepreneurs (Guterresa et al., 2020). Economic capital, human capital, and social capital have always been the three traditional capital to improve the efficiency of enterprises. However, with the rapid development of the world economy, more and more entrepreneurs have realized that it is far from enough to rely only on capital development in the traditional sense. Accordingly, the development of different leadership styles guided by entrepreneurs’ psychological capital has become another important way to enhance the competitiveness of enterprises (Boudreaux and Nikolaev, 2019; Honig and Hopp, 2019; Stevenson et al., 2019). In addition, the improvement of organizational learning ability is the most promising way for an enterprise to maintain its current and future competitive advantages. To build an enterprise into a learning organization is a necessary condition for its sustainable development and growth in the future. In general, organizational learning involves the relationship between the enterprise and the external environment, and the goal is to adapt to the changes in the entrepreneurial environment and obtain corresponding competitive advantages (D’Angelo and Presutti, 2019). Therefore, it is crucial to improve the organizational performance and learning ability of enterprises.

As a leader in the process of enterprise development, the development of entrepreneur’s physical, and mental quality is also crucial. A good physical quality can enable entrepreneurs to cope with changes in the external environment and internal pressure and keep working (Abubakar, 2019; Nasir and Anwar, 2019; Rahman et al., 2019). Psychological quality is more important, including leadership style, temperament type, psychological state, and pressure bearing (Namisi et al., 2019). Many relevant studies have found that the leadership styles of entrepreneurs with different types of entrepreneurial psychology are diverse (Sözbilir, 2019). Lau et al. (2020) proposed the transformational psychological leadership style and entrepreneurial psychological leadership style and analyzed and found that the transformational psychological leadership style had particularity, complexity, and certain risks, so it was necessary to coordinate the internal and external relations of the enterprise. Sandybayev (2019) conducted a quantitative analysis of 87 small- and medium-sized enterprise (SME) participants in the United Arab Emirates and concluded that the entrepreneurial leadership style had more opportunities to effectively manage the organization than just a manager or just an informal leader without status authority, which might improve the organizational performance of the enterprise. Different entrepreneurial psychological leadership styles may have different effects on the organizational learning ability and organizational performance of enterprises, but a series of influence situations still need to be studied (Tlaiss and Kauser, 2019).

To sum up, there are many reports on leadership styles caused by different psychological qualities, but few studies on the influence of leadership styles on organizational performance and learning ability. Based on this, 421 general managers, middle and senior managers, grassroots managers, and ordinary employees of 350 private SMEs in the Beijing region are selected as research objects. The impact of the entrepreneurial psychological leadership style on organizational learning ability and organizational performance is comprehensively evaluated through the construction of a hypothesis model of leadership style and organizational learning ability and organizational performance and questionnaire survey (Zheng et al., 2015).

## Literature Review

At present, the research on the relationship between entrepreneurs’ own leadership ability and enterprise performance has attracted the attention of many scholars. Sawaean and Ali (2020) and Yu et al. (2019) attempted to investigate the determinants of entrepreneurial leadership in the organizational performance of 500 SMEs in Kuwait. The results showed that entrepreneurial leadership and total quality management practices had a beneficial and considerable impact on organizational performance. The relationship between entrepreneur leadership and SME organizational performance was basically regulated by total quality management practices. Due to the differences in individual factors, the performance of leadership of different entrepreneurs at the psychological level was also varied. Pirayesh and Pourrezay (2020) proposed the need for an appropriate leadership style to regulate and improve organizational culture in order to improve environmental performance. The partial least square method was used to analyze the population of 317 statistical samples, and it was found that transformational psychological leadership had a greater impact on enterprise innovation than charismatic leadership. Yamin (2020) proposed that managers and policy makers should consider intrinsic motivation and transformational leadership in order to improve employee retention and organizational performance through effect scale analysis. The improvement of organizational learning ability is an important factor related to the future development of enterprises. The influence of leaders on their development has also been explored by many scholars. Darwish et al. (2020) found that transformational leadership can adjust the relationship between exploratory and transformational learning processes and innovation based on the investigation and analysis in the United Arab Emirates and enhance enterprise learning. Ricardianto et al. (2020) used sampling techniques to analyze the influence of leadership style, work–life balance, and employee engagement on the work efficiency of 290 crew members. The results showed that leadership style, work–life balance, and employee engagement had a direct and positive effect on work performance. Dewi and Wibow (2020) used multiple linear regression to analyze the job performance of university teachers. The study found that style of instruction, organizational culture, and motivation influenced both learning and work performance of long-term lecturers. Arokiasamy and Tat (2020) conducted an empirical study on 369 full-time academic employees of higher-education institutions in Malaysia. The results showed that transformational leaders were able to create a workplace spirit that increased employees’ ability to engage in learning (Chen et al., 2019).

To sum up, the current research on different psychological leadership styles is a hot topic, and there are many researches on enterprise performance and individual learning input of employees, but there are few researches on the influence mechanism of entrepreneurial leadership styles, organizational learning, and enterprise performance (Gross and Cabanda, 2016). Therefore, the AMOS 17.0 software is used to study the role of entrepreneurs with different psychological leadership styles in organizational learning and organizational performance.

## Methodology

### Research Subjects

In this study, 421 general managers, middle managers, and grassroots managers of 350 private SMEs in Beijing were selected as research objects. Questionnaires were distributed online and on-site. A total of 530 copies of the scale were distributed, including 110 copies in electronic form and 420 copies in paper form. There were 495 recycling scales, including 86 in electronic form and 409 in paper form. After deletion of invalid data, there were 467 valid scales, with an effective rate of 88.11%.

**FIGURE 1:**
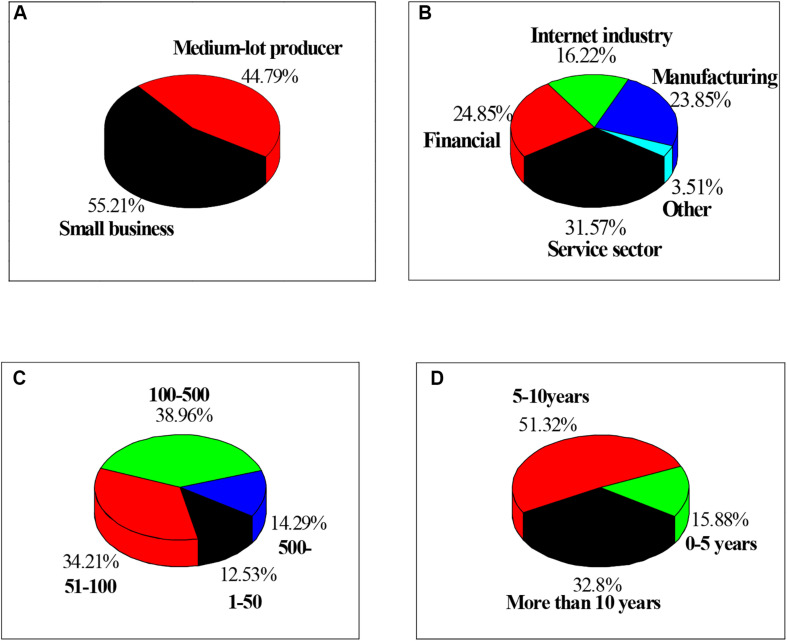
Basic information of all tested enterprises. Note: **(A)** is the scale of the enterprise; **(B)** is the industry to which the enterprise belongs; **(C)** is the number of employees; and **(D)** is the establishment time of the enterprise.

The selected enterprise is shown in Figure 1. There are 193 small enterprises, accounting for 55.2%; 157 medium-sized enterprises, accounting for 44.79%; and 110 service enterprises, accounting for 31.57%. Besides, there are 83 enterprises in the financial sector, accounting for 24.85%; 57 enterprises in the Internet sector, accounting for 16.22%; 83 enterprises in the manufacturing sector, accounting for 23.85%; and 12 enterprises in other industries, accounting for 3.51%. There are 44 enterprises with 1–50 employees, accounting for 12.53% of the total; 120 enterprises with 51–100 employees, accounting for 34.21%; 136 enterprises with 100–500 employees, accounting for 38.96%; and 50 enterprises with more than 500 employees, accounting for 14.29%. In addition, 55 enterprises are established in 0–5 years, accounting for 15.88%; 180 enterprises in 5–10 years, accounting for 51.32%; and 115 enterprises over 10 years, accounting for 32.8%.

**FIGURE 2:**
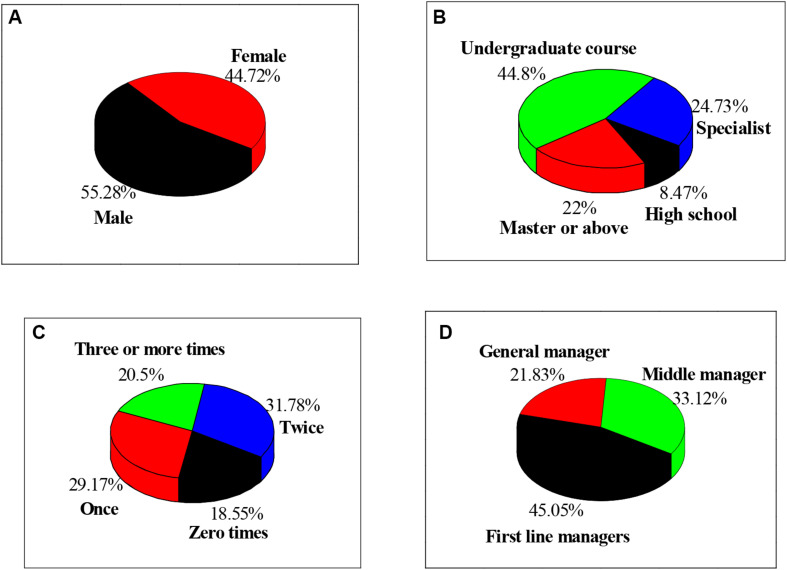
Basic information of all subjects. Note: **(A)** is the gender of the subjects; **(B)** refers to the educational background of the subjects; **(C)** is the number of entrepreneurships of the subjects; and **(D)** is the position of the subject.

The selected subjects are shown in Figure 2. There are 233 males, accounting for 55.28%, and 188 females, accounting for 44.72%. Furthermore, there are 36 subjects with a high school education background or below, accounting for 8.47%, and 104 subjects with a college education background, accounting for 24.73%. Among them, there are 189 people with bachelor’s degrees, accounting for 44.8%, and 93 with master’s degrees or above, accounting for 22%. A total of 78 of the subjects have no entrepreneurial experience, accounting for 18.55%. The number of entrepreneurs is 1,123, accounting for 29.17%. Meanwhile, there are 134 people who have started business twice, accounting for 31.78%, and 86 people who have started business three times or more, accounting for 20.5%. Among the subjects, 92 are general managers, accounting for 21.83%; 139 middle managers, accounting for 33.12%; and 190 managers at the grassroots level, accounting for 45.05%.

### Research Hypothesis

Each entrepreneur has a different leadership style because of their own different living environment, temperament, and psychological quality. The transformational psychological leadership style can clearly identify the need for innovation, which is of great help to the innovation and reform of enterprises and thus conducive to the improvement of organizational learning ability and performance. The transactional psychological leadership style generally adopts the method of reward to encourage employees to work actively and hard and at the same time guides the enterprise’s innovation atmosphere, which is conducive to the long-term promotion of organizational learning and performance. The laissez-faire psychological leadership style does not manage employees too much, allows them to work according to their own ideas and ways, and provides enough space and freedom. In the long run, it can also promote the organizational learning ability of employees. Therefore, the following hypotheses are proposed in the study.

I. Leadership style and organizational performance
  S1: There is a positive relationship between leadership style and organizational performance.
    S1a1: There is a positive relationship between the transformational psychological leadership style and financial performance.
    S1a2: There is a positive relationship between the transformational psychological leadership style and growth performance.
    S1b1: The transactional psychological leadership style has a positive relationship with financial performance.
    S1b2: There is a positive relationship between the transactional leadership style and growth performance.
    S1c1: The laissez-faire psychological leadership style has a positive relationship with financial performance.
    S1c2: The laissez-faire psychological leadership style has a positive relationship with growth performance.

II. Leadership style and organizational learning
  P1: There is a positive relationship between leadership style and organizational learning ability.
    P1a1: There is a positive relationship between the transformational psychological leadership style and organizational learning ability.
    P1b1: The transactional psychological leadership style has a positive effect on organizational learning ability.
    P1c1: The laissez-faire psychological leadership style has a positive relationship with organizational learning ability.

### The Relationship Model Between Leadership Style and Organizational Learning Ability and Organizational Performance

**FIGURE 3:**
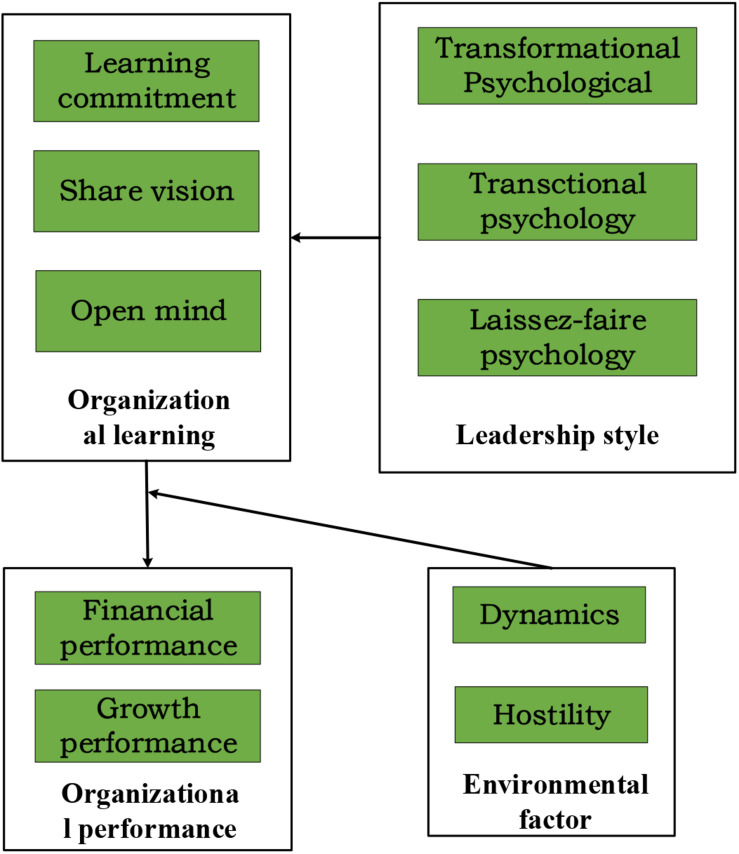
The relationship model between the entrepreneurial psychological leadership style and organizational learning ability and organizational performance.

In empirical studies, the relationship model between leadership style and organizational learning ability and organizational performance as shown in Figure 3 is usually constructed based on the above assumptions. The independent variables of this study are leadership style, including the transformational psychological leadership style, transactional psychological leadership style, and laissez-faire psychological leadership style. The dependent variables are organizational performance and organizational learning ability, among which organizational performance includes organizational financial performance and organizational growth performance and organizational learning includes learning commitment, sharing vision, and open-mindedness. Based on the background of dynamic and hostile entrepreneurial environment, different entrepreneurial psychological leadership styles can positively affect organizational performance and organizational learning ability.

### Questionnaire Design

In empirical research, a questionnaire is usually selected to conduct research and obtain a large amount of data that need to be known in the research. These data are not expensive to collect, describe the problem well, and have a certain degree of reliability. Because of this, a questionnaire survey is favored by many researchers. Through a well-researched and well-designed questionnaire, it is possible to have an overall understanding of the object under investigation, and at the same time, it is possible to have a specific understanding of each part of the target object. The questionnaire survey has become one of the most commonly used methods to obtain information. Therefore, according to the theoretical hypothesis and relationship model proposed in this study, the variables to be measured by scale tools were identified as leadership style, organizational performance, and organizational learning ability. Finally, social entrepreneurship psychology leadership style scale, organizational performance scale, and organizational learning ability scale were selected.

#### Entrepreneurial Psychology Leadership Style Scale

As shown in Table 1, the questionnaire includes the transformational psychological leadership style, transactional psychological leadership style, and laissez-faire psychological leadership style. Richter’s five points are used for each item: 1 for “strongly disagree,” 2 for “disagree,” 3 for “neither agree nor disagree,” 4 for “agree,” and 5 for “strongly agree.” The internal consistency reliability values of three dimensions of the transformational psychological leadership style, transactional psychological leadership style, and laissez-faire psychological leadership style are 0.82, 0.87, and 0.91, respectively.

**TABLE 1:** Different entrepreneurial psychological leadership style scales.

**Variables**	**Measurement items**
Transformational psychological	The leader has a clear understanding of the company’s prospects
leadership style	Leaders encourage employees to see the changing environment as an opportunity
	The leader has a clear understanding of the department’s development direction in 5 years
	Leaders inspire employees to think about old problems in new ways
Transactional	Leaders tell employees what to do
psychological leadership style	The expectations and rewards that leaders give to their employees
	The employee can negotiate with the leader about the content of the employee’s work
	The leader only asks employees about work
	Leaders discourage employees from taking the initiative
Laissez-faire	Avoiding getting sucked into major problems
psychological	Not present when needed
leadership style	Avoiding making decisions
	Delaying response to urgent problems

#### Organizational Performance Scale

As shown in Table 2, the scale includes two dimensions of organizational financial performance and organizational growth performance. Organizational financial performance includes three measurement items, and organizational growth performance includes three measurement items. Richter’s five points are used for each item: 1 for “strongly disagree,” 2 for “disagree,” 3 for “neither agree nor disagree,” 4 for “agree,” and 5 for “strongly agree.” After design, the internal consistency reliability values of the overall scale, organizational financial performance and organizational growth performance, are 0.84 and 0.86, respectively.

**TABLE 2:** Organization performance scale.

**Variables**	**Measurement items**
Organization financial	The main business of enterprises has always occupied a high market share
performance	Corporate net profit margins have remained high
	The enterprise ROE occupies the good advantage in the peer enterprise
Organization growth	Compared with the peer enterprises, the sales quota of enterprises has increased rapidly
performance	Compared with the peers, the net profit margin of the enterprise has increased rapidly
	Compared with the peer enterprises, the product update speed and service development are rapid

#### Organizational Learning Ability Scale

As shown in Table 3, the scale includes three dimensions: learning commitment, sharing vision, and open mind. The learning commitment includes five measurement items, the sharing vision five measurement items, and the open mind five measurement items. Richter’s five points are used for each item: 1 for “strongly disagree,” 2 for “disagree,” 3 for “neither agree nor disagree,” 4 for “agree,” and 5 for “strongly agree.” After design, the internal consistency reliability values of the three dimensions of the scale, namely, learning commitment, sharing vision, and open mind, are 0.90, 0.83, and 0.92, respectively.

**TABLE 3:** Organization learning ability scale.

**Variables**	**Measurement items**
Learning commitment	The management believes that the learning ability of the enterprise is the competitive advantage of the enterprise
	The company regards learning as one of the core values for future development and improvement
	The company sees learning as an investment rather than a cost
	The enterprise sees active learning as a necessary quality for future survival
	The enterprise does not attach importance to the learning of employees
Sharing vision	Employees have a clear understanding of the company’s future development direction and positioning
	The personnel of any department in this enterprise have the vision of organization and unity
	All the staff of the enterprise try their best to accomplish the enterprise’s goals
	Management shares their vision with employees
	The company does not have a clear vision
Open mind	Employees are not afraid to question the company’s operating strategy
	The management does not like its views to be questioned
	It is believed in the enterprise that everyone should accept and accommodate different voices
	The management of the enterprise encourages the staff to actively innovate and think
	The enterprise values originality very much

### Analysis Method

SPSS 19.0 was used to process the data in this study. The counting data were expressed as a percentage (%). Multiple regression models were used to analyze the correlation between transformational, transactional, and laissez-faire psychological leadership styles and organizational financial performance, growth performance, and learning ability. Furthermore, AMOS 7.0 software was used to simulate and verify the relationship between different entrepreneurial psychological leadership styles and organizational learning ability and organizational performance, with Origin 7.5 mapping.

## Results

### Differences of Varied Entrepreneurial Psychological Leadership Styles in Demographics

**FIGURE 4:**
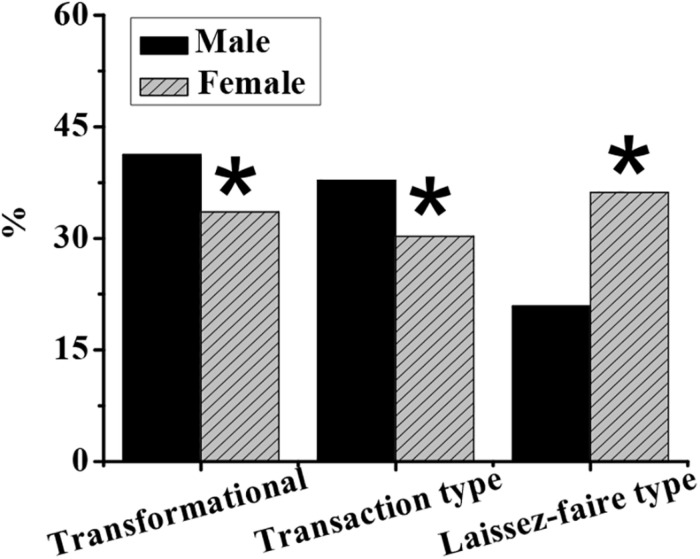
Gender differences in different entrepreneurial psychological leadership styles. Note: An asterisk (*) indicates that the difference is statistically significant compared to males (*p* < 0.05).

**FIGURE 5:**
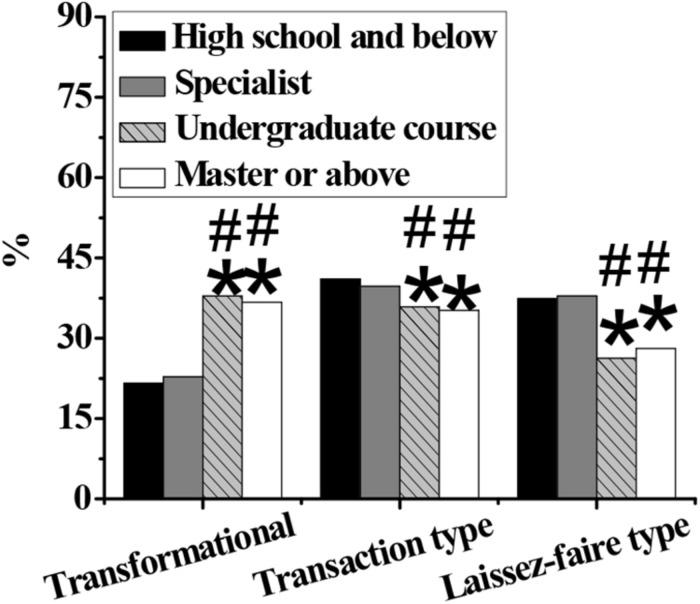
Differences in educational background between different entrepreneurial psychological leadership styles. Note: An asterisks (*) indicates that there is a statistically significant difference (*p* < 0.05) compared with high school degree and below; A number sign (^#^) indicates that the difference is statistically significant compared with the junior college degree (*p* < 0.05).

**FIGURE 6:**
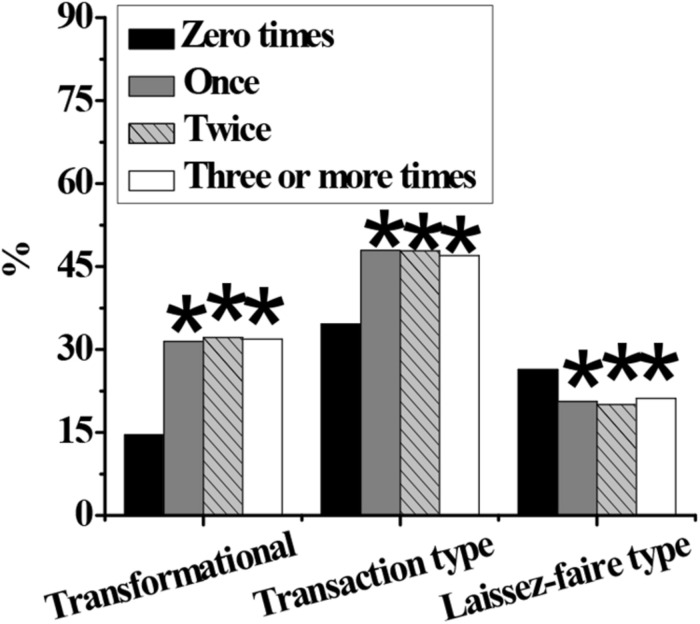
Difference in entrepreneurial times of different entrepreneurial psychological leadership styles. Note: An asterisk (*) indicates that there is a statistically significant difference compared with zero start-up (*p* < 0.05).

**FIGURE 7:**
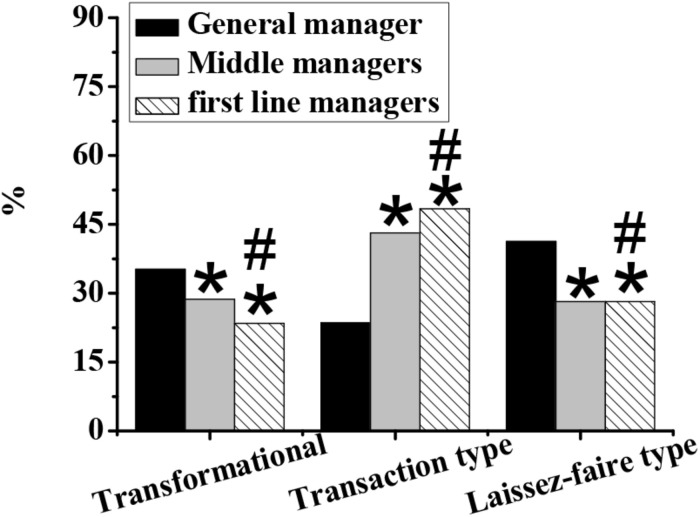
The differences of different entrepreneurial psychological leadership styles in positions. Note: An asterisk (*) indicates that the difference is statistically significant compared to general managers (*p* < 0.05); a number sign (#) indicates that the difference is statistically significant compared with middle managers (*p* < 0.05).

As shown in Figures 4–7, in terms of gender, the proportion of transformational and transactional psychology in males is significantly higher than that in females, while the proportion of laissez-faire psychology is significantly lower than that in females (*p* < 0.05). In terms of educational background, the proportion of transformational psychology of people with a bachelor’s degree, master’s degree, or above is significantly higher than that with junior college degree or below, and the proportion of transactional and laisses-faire psychology is significantly lower than that with junior college degree or below (*p* < 0.05). In terms of the number of start-ups, the proportion of transformational, and transactional psychology of zero start-ups is significantly lower than that of one, two, and more than three start-ups, and the proportion of laissez-faire psychology is significantly higher than that of one, two, and more than three start-ups (*p* < 0.05). In terms of position, the proportion of general managers’ transformational, and laissez-faire psychology is significantly higher than that of middle managers and grassroots managers, and the proportion of transactional psychology is significantly lower than that of middle managers and grassroots managers (*p* < 0.05).

### Analysis on the Correlation Between the Entrepreneurial Psychological Leadership Style and Organizational Performance

As shown in Table 4, the leadership style of entrepreneurial psychology is taken as the independent variable and organizational performance as the dependent variable. A unary linear regression analysis is carried out. The standardized regression coefficient between the entrepreneurial psychological leadership style and organizational performance is 0.565, showing a significant positive correlation (*p* < 0.05). Thus, assumption S1, “there is a positive relationship between leadership style and organizational performance,” is true.

**TABLE 4:** Analysis on the correlation between the entrepreneurial psychological leadership style and organizational performance.

**Models**	**Non-standardized coefficient**		**Standardized coefficient**	***t***	***p***
	***B***	**Standard error**	**Beta**		
Constant term	15.271	3.016		3.192	0.028
Leadership style	0.433	0.117	0.565	4.638	0.011

As shown in Table 5, taking the transformational psychological leadership style, transactional psychological leadership style, and laissez-faire psychological leadership style as independent variables and organizational financial performance as dependent variable, a multifactor regression analysis is conducted. The standardized regression coefficient of the transformational psychological leadership style and organizational financial performance is 0.384, showing a very significant positive correlation (*p* < 0.001). Thus, assumption S1a1, “transformational psychological leadership style has a positive relationship with financial performance,” is true. The standardized regression coefficient between the transactional psychological leadership style and organizational performance is 0.372, showing a significant positive correlation (*p* < 0.05). Thus, assumption S1b1, “transactional psychological leadership style has a positive relationship with financial performance,” is true. There is no significant positive correlation between leadership style and organizational performance (*p* > 0.05). Thus, assumption S1c1, “laissez-faire psychological leadership style has a positive relationship with financial performance,” is not true.

**TABLE 5:** Multiple regression analysis of leadership style of different entrepreneurial psychology styles and organizational financial performance.

**Models**	**Non-standardized coefficient**		**Standardized coefficient**	***t***	***p***
	***B***	**Standard error**	**Beta**		
Constant item	12.782	1.495		6.776	0.008
Transformational psychology	0.316	0.087	0.384	3.822	0.000
Transactional psychology	0.348	0.093	0.372	4.517	0.002
Laissez-faire psychology	0.201	0.131	0.250	1.660	0.065

As shown in Table 6, taking the transformational psychological leadership style, transactional psychological leadership style, and laissez-faire psychological leadership style as independent variables and organizational growth performance score as dependent variable, a multifactor regression analysis is conducted. The standardized regression coefficient of the transformational psychological leadership style and organizational growth performance is 0.630, showing an extremely significant positive correlation (*p* < 0.001). Thus, assumption S1a2, “there is a positive relationship between transformational psychological leadership style and growth performance,” is true. The standardized regression coefficient between the transactional leadership style and organizational growth performance is 0.463, showing a significant positive correlation (*p* < 0.05). Thus, hypothesis S1b2, “there is a positive relationship between transactional leadership style and growth performance,” is true. The standardized regression coefficient between the laissez-faire psychological leadership style and organizational growth performance is 0.357, and there is no significant positive correlation (*p* > 0.05). Thus, assumption S1c2, “laissez-faire psychological leadership style has a positive relationship with growth performance,” is not true.

**TABLE 6:** Regression analysis of the entrepreneurial leadership style and organizational growth performance.

**Models**	**Non-standardized coefficient**		**Standardized coefficient**	***t***	***p***
	***B***	**Standard error**	**Beta**		
Constant item	13.339	3.174		8.492	0.005
Transformational psychology	0.572	0.107	0.630	6.211	0.000
Transactional psychology	0.428	0.085	0.463	5.380	0.003
Laissez-faire psychology	0.416	0.099	0.357	4.827	0.057

### Analysis on the Correlation Between the Entrepreneurial Psychological Leadership Style and Organizational Learning Ability

As shown in Table 7, a unary linear regression analysis is carried out with the entrepreneurial psychology leadership style as the independent variable and organizational learning ability as the dependent variable. The standardized regression coefficient between the entrepreneurial psychological leadership style and organizational learning ability is 0.695, showing a significant positive correlation (*p* < 0.05). Thus, hypothesis P1, “there is a positive relationship between leadership style and organizational learning ability,” is true.

**TABLE 7:** Regression analysis of the entrepreneurial psychological leadership style and organizational learning ability.

**Models**	**Non-standardized coefficient**		**Standard coefficient**	***t***	***p***
	***B***	**Standard error**	**Beta**		
Constant item	11.421	4.618		5.374	0.017
Leadership styles	0.632	0.126	0.695	5.792	0.012

As shown in Table 8, taking the transformational psychological leadership style, transactional psychological leadership style, and laissez-faire psychological leadership style as independent variables and organizational learning ability as dependent variable, a multivariate regression analysis is conducted. The standardized regression coefficient of the transformational psychological leadership style and organizational learning ability is 0.573, showing an extremely significant positive correlation (*p* < 0.001). Thus, hypothesis P1a1, “there is a positive relationship between the transformational psychological leadership style and organizational learning ability,” is true. The standardized regression coefficient between the transactional psychological leadership style and organizational learning ability is 0.564, showing a significant positive correlation (*p* < 0.05). Thus, assumption P1b1, “transactional psychological leadership style has a positive effect on organizational learning ability,” is true. The standardized coefficient of the laissez-faire psychological leadership style and organizational learning ability is 0.398, showing a significant positive correlation (*p* < 0.05). Thus, assumption P1c1, “laissez-faire psychological leadership style has a positive relationship with organizational learning ability,” is true.

**TABLE 8:** Regression analysis of different entrepreneurial psychology leadership style and organizational learning ability.

**Models**	**Non-standardized coefficient**		**Standardized coefficient**	***t***	***p***
	***B***	**Standard error**	**Beta**		
Constant item	14.190	3.881		4.728	0.000
Transformational psychology	0.482	0.063	0.573	5.295	0.000
Transactional psychology	0.515	0.082	0.564	4.896	0.008
Laissez-faire psychology	0.362	0.071	0.398	4.184	0.011

### Simulation and Verification of the Relationship Model of Different Entrepreneurial Psychology Leadership Styles, Organizational Learning Ability, and Organizational Performance

**FIGURE 8:**
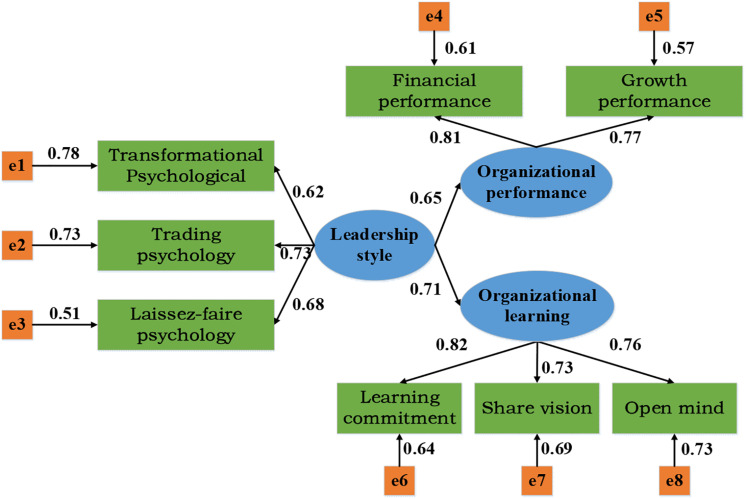
The structural equation path analysis of the relationship model between the different entrepreneurial psychological leadership styles, organizational learning ability, and organizational performance.

As shown in Figure 8, firstly, AMOS 17.0 was used to obtain the structural equation path analysis of the relationship model between different entrepreneurial psychological leadership styles, organizational learning ability, and organizational performance. The covariance between the error terms of the external cause potential variables and the covariance between the error terms of the internal cause potential variables are both positive, and the factor load range of each potential variable and the observed variable is 0.5–1, basically meeting the requirements of the model.

Then the external quality of the model was evaluated. Table 9 shows the key fitting indexes of the relationship model. The absolute fitting statistics of the relationship model (*X*^2^ fitting priority index, approximation of root mean square error, goodness of fit test, and cross-validity test index), value-added fitness statistics (normative fit index, value-added fit index, comparative fit index, and Tuckers–Lewis index), and reduced fitness statistics (CAIC, NNFI, and AGFI) all meet the corresponding fitness standards. The model basically fits the data.

**TABLE 9:** The fitting index of the relationship model between leadership style and organizational learning ability and organizational performance.

**Measurement items**	**Fitting index**	**Fit criteria**	**Fit results**
**Absolute fitness statistics**			
*X*^2^ fitting priority index	2.481	<3.10	Fitting
Approximation of root mean square error	0.066	<0.08	Fitting
Goodness of fit test	1.042	>0.95	Fitting
Cross validity test index	0.657 < 0.815	theoretical model value < independent value	Fitting
	0.657 < 5.712	theoretical model value < saturation value	Fitting
Value-added fitness statistics			
Normative fit index	1.037	>0.95	Fitting
Value-added fit index	0.972	>0.95	Fitting
Comparative fit index	0.996	>0.95	Fitting
Tuckers–Lewis index	1.053	>0.95	Fitting
**Reduced fitness statistics**			
CAIC	273.67 < 517.44	theoretical model value < independent value	Fitting
	273.67 < 1527.70	theoretical model value < saturation value	Fitting
NNFI	0.632	>0.50	Fitting
AGFI	0.711	>0.50	Fitting

## Discussion

As a leader in the operation of an enterprise, the leadership style brought by the entrepreneur’s physical and mental qualities directly affects the future development status and direction of the enterprise, and the adjustment mechanism of organizational performance by the entrepreneur’s leadership style needs to be further studied. Therefore, the questionnaire survey and other empirical research are conducted on SMEs in the Beijing region. First, it is found that the proportion of transformational and transactional psychology in men is significantly higher than that in women and that the proportion of laissez-faire psychology is significantly lower than that in women (*p* < 0.05), which is consistent with the study results of Miao et al. (2019), indicating that men are better at applying and learning new things, while women are better at exerting employees’ autonomy. The proportion of transformative psychology of people with a bachelor’s degree or above is significantly higher than that with a junior college degree or less, and the proportion of transactional and laissez-faire psychology is significantly lower than that with a junior college degree or less (*p* < 0.05), which is different from the results of Palmer et al. (2019) on the difference between the leadership style of doctoral and undergraduate degrees. The possible reason is that the sample size of doctors involved in this study is too small, resulting in some deviation to the results. The proportion of transformational and transactional psychology in people with zero entrepreneurship is significantly lower than that in people with one or more entrepreneurships (*p* < 0.05), indicating that people with entrepreneurial experience may have more management experience and know how to encourage employees with rewards. The proportion of general managers’ transformational and laissez-faire psychology is significantly higher than that of middle and grassroots managers (*p* < 0.05), which is the same as the research results of Digan et al. (2019). Managers at the general manager level are mainly responsible for making decisions on changes in the general direction of the company and encouraging employees to be proactive and innovative. The results show that the standardized regression coefficient of entrepreneurial psychological leadership style and organizational performance is 0.265, showing a significant positive correlation (*p* < 0.05), which is consistent with the research results of Jafari-Moghadam et al. (2020), which indicates that the leadership style has a certain positive effect on organizational performance, and hypothesis S1 is verified. The correlation between different leadership style dimensions and organizational performance is analyzed. The transformational psychological leadership style is positively correlated with organizational financial performance and growth performance (*p* < 0.001), while the transactional psychological leadership style is positively correlated with organizational financial performance and growth performance (*p* < 0.05), which is basically consistent with the research results of Newman et al. (2018), which show that both the transformational psychological leadership style and transactional psychological leadership style can have a positive effect on organizational performance but that the transformational psychological leadership style has a higher impact than the transactional psychological leadership style. This may be due to the fact that the transformational psychological leadership style is more prominent in leadership effectiveness, job satisfaction, and organizational commitment (Sutanto, 2017; Abubakar, 2019). However, there is no significant positive correlation between the laissez-faire psychological leadership style and organizational performance (*p* > 0.05), which indicates that the laissez-faire psychological leadership style cannot play a positive role in promoting the future development of enterprises.

Organizational learning ability is a key source for enterprises to obtain and maintain competitive advantages in the future (Ning and Li, 2018). Therefore, the influence of entrepreneurial leadership style on organizational learning ability is analyzed. It is found that the transformational psychological leadership style and transactional psychological leadership style are positively correlated with organizational learning ability (*p* < 0.001); there is a significant positive correlation between the laissez-faire psychological leadership style and organizational learning ability (*p* < 0.05), which is consistent with the research results of Peris-Ortiz et al. (2018), which shows that the three different psychological leadership styles can promote the improvement of organizational learning ability, but the transformational psychological leadership style and the transactional psychological leadership style have a higher influence than the laisse-faire psychological leadership (Tajeddini et al., 2017). In addition, AMOS 17.0 software is used to simulate and verify the relationship model between entrepreneurial psychological leadership style, organizational learning ability, and organizational performance established in this study. The results show that the covariance between the error terms of the internal and external factor potential variables is positive, and the factor load value of each potential variable and the observed variable is between 0.5 and 1. Moreover, the absolute fitness statistics, value-added fitness statistics, and reduced fitness statistics of the model all meet the corresponding fitness standards, which is basically consistent with the research results of Penger and Grah (2019; Wu and Wu, 2019), which shows that the model presented in this study meets the basic requirements and is feasible.

## Conclusion

The influence of different entrepreneurial psychological leadership styles on organizational learning ability and organizational performance was analyzed by establishing a hypothesis model of the relationship between leadership style and organizational learning ability and organizational performance, providing experimental basis for the improvement of enterprise learning and performance in the future. However, only enterprises in the Beijing region were selected as research objects, bringing about certain regional limitations. In addition, there is also a certain relationship between organizational learning ability and organizational performance, but there is no further analysis about it. In the future, it is advised to increase the sample size to study the relationship between organizational learning and performance. In a word, the transformational psychology and transactional psychological leadership styles have a more positive influence on organizational performance and learning ability than the laissez-faire psychological leadership style. The hypothesis model of the relationship between the entrepreneurial psychological leadership style, organizational learning ability, and organizational performance conforms to the adaptation criteria and is feasible.

## Data Availability Statement

The raw data supporting the conclusions of this article will be made available by the authors, without undue reservation, to any qualified researcher.

## Ethics Statement

The studies involving human participants were reviewed and approved by Tsinghua University Ethics Committee. The patients/participants provided their written informed consent to participate in this study.

## Author Contributions

YT wrote the manuscript and made the validation all by himself.

## Conflict of Interest

The author declares that the research was conducted in the absence of any commercial or financial relationships that could be construed as a potential conflict of interest.
